# Abstractness emerges progressively over the second year of life

**DOI:** 10.1038/s41598-022-25426-5

**Published:** 2022-12-03

**Authors:** Francesca Bellagamba, Anna M. Borghi, Claudia Mazzuca, Giulia Pecora, Fabiana Ferrara, Alan Fogel

**Affiliations:** 1 Department of Dynamic and Clinical Psychology, and Health Studies, Sapienza University of Rome, Rome, Italy; 2 Institute of Cognitive Sciences and Technologies, Italian National Research Council, Rome, Italy; 3 Department of Psychology, University of Utah, Salt Lake City, USA

**Keywords:** Psychology, Health care

## Abstract

Abstract words, terms not referring to here and now, are acquired slowly in infancy. They are difficult to acquire as they are more detached from sensory modalities than concrete words. Recent theories propose that, because of their complexity, other people are pivotal for abstract concepts’ acquisition and use. Eight children (4 girls) and their mothers were observed longitudinally and extensively from 12 to 24 months of age. Video recordings of mother-infant free play with toys were done every two weeks in a laboratory setting with families in the USA. Children progressively use a range of words referring to abstract concepts, with a major shift from 12 to 15 months and again from 22 to 24 months, but the qualitative data testify an incremental growth of abstract concepts. We identified a progression in the acquisition of words denoting abstract concepts in relation to the overall productive vocabulary, suggesting that having more abstract terms in one’s vocabulary promotes faster language acquisition.

## Introduction

Research on developmental change based on a dynamic systems perspective^1,2^ defines communication frames as regularly occurring patterns that regulate the communication and sharing between the participants, such as co-action episodes, social games, pretending, and talking about past experience. A microgenetic approach, in which individuals are observed through a period of developmental change and observations are conducted before, during, and after a period of developmental change, can be extremely useful to understand the change processes in development^3^. In this study, we consider the transition to symbolic communication by examining changes in communication frames in which mother and child jointly participate in the activity of playing with objects and how infants progress from communicating on concrete objects to communicating using more abstract concepts.

Several authors have emphasized the importance of communication frames involving parent-infant play for establishing joint reference, and play is considered a fertile condition through which early language is facilitated and acquired^4,5^. By participating in social games with parents, infants also gradually develop a sense of self that is initially totally embodied and that gradually develops to include symbols, imagination, and language by the end of the second year^6–8^. Symbolic play in particular requires the establishment of collective intentionality and the necessity to negotiate meaning^5^.

In recent years a different research stream, focusing on abstract concepts (e.g., “fantasy”), has emphasized the role of social interaction to develop conceptual knowledge and language acquisition. Barsalou and colleagues^9,10^ underlined how introspection and the social aspects of situations characterize more abstract than concrete concepts. Multiple representation theories have underlined the crucial role not only of sensorimotor but also of linguistic, social, and emotional experiences for the formation and representation of abstract concepts (review in^11^; see^12,13^). Within these theories, the Words as Social Tools (WAT) approach has ascribed a special role to social interaction^14,15^. Compared to words referring to concrete concepts (e.g., “spoon”), abstract words like “friend” and “idea” are generally more complex: they typically do not have a single object as a referent but evoke complex scenes and situations^16,17^. Furthermore, they are generally acquired later and through linguistic rather than perceptual modalities, i.e., by verbally explaining their content rather than pointing to their referent (Modality of Acquisition^18^), are less iconic—that is—associated to a specific referent through a figurative-symbolic relationship—than concrete words^19^, and are generally relational^20^. Abstract-symbolic words require a shared understanding which grows, according to dynamical system theories, via the development of social play communication frames.

Crucially for us, more abstract concepts collect more heterogeneous exemplars than concrete ones (low dimensionality^21^): for example, whereas the members of the concrete category “ball” are perceptually similar, the members of the abstract category “freedom” are very different. According to the WAT proposal, abstract concepts would be more grounded in language developed in shared communication frames than concrete concepts^12,14,22^: because of the heterogeneity of their members, we would need other people and frames with different themes to acquire them and understand their meaning^23–25^. Notice that we deem social interaction important both for the acquisition of concrete and abstract concepts, but we contend that its role might be particularly critical for the latter. To clarify: the meaning of a concept like “freedom” might elicit situations as diverse as “running on the grass”, “escaping from prison”, and “flying in the sky”. Thus, the contribution of other people who use the same label across the various communication frames (situations) and eventually explain to us the word meaning is crucial to acquire and comprehend abstract concepts like “freedom”, more than concrete concepts like “ball”. Hence, other people are crucial to acquire abstract words, because explanations from others and linguistic labels are very helpful to put together a variety of heterogeneous experiences, situations, etc. In addition, different people will have different perspectives on the meaning of abstract words. This means that the child must make mental inferences, taken from different social situations, to create or construct a mental concept of, for example, “freedom”. Also, that concept will continue to evolve with different instances as it comes up in discussions. So, what makes something abstract is that it involves “going beyond the information given” and thus the construction of a mental life that goes beyond the concrete world and leads to invention, imagination, creative thinking, inferential thought, curiosity, etc. Notably, not only abstract concepts collect heterogeneous exemplars, but abstract concepts are heterogeneous themselves since they belong to different kinds, from emotions to numerical concepts, as many recent studies on adults have highlighted^26–29^. Hence, the dimensions we have mentioned might assume a different weight depending on the considered conceptual kind.

Despite an emergent interest in a special relationship between abstract concepts and social interaction, the evidence on the relevance of social interaction for abstract concepts acquisition is limited to only a few studies with infants. Parents use abstract vocabulary even during the first year of life^30^. An experiment on early development showed, however, that comprehension of infants’ first abstract concepts occurs between 10 and 14 months, in parallel with the emergence of social communication abilities like gaze following, and joint action^31^. These results suggest a strict interrelation between abstract concepts acquisition and specific kinds of social interaction. An important question to be raised is, in our view, how social interaction creates the conditions for abstract thinking. Both gaze following and joint action infer shared mental states that are not, at the outset, defined concretely. When we follow someone’s gaze, we don’t “know” where that will lead in the same way that we “know” a concrete object to which a gesture like pointing refers. So this “not knowing” creates a kind of mental “space” of possibility that is the beginning of abstract mental spaces.

From developmental research—using diaries (e.g.^32^), maternal interviews (e.g.^33^), maternal written reports^34^, systematic observations (e.g.^35,36^), and large repository data from parent-report instruments^37^—we know that between 10 and 13 months of age most infants begin to produce their first words. They reach the milestone of a 10-word productive vocabulary between 13 and 19 months^34,38^. Early words belong to a variety of linguistic categories. Generally, they consist of names for caregivers (e.g. *mama*, *dad*, *grandma*), small and concrete objects the infant can manipulate and act upon (e.g. *duck*, *shoe*, *teddy*, *spoon*), words used to regulate social interaction (e.g. *no*, *more*, *bye*), and cognitive-relational words encoding abstract concepts such as failure, disappearance, recurrence, and location (e.g. *gone*, *more*, *there*)^34,39^. Some words are acquired in a social-routine context and later are extended to new contexts. For example, labels for small concrete objects may be initially acquired through associative learning during repeated ‘give and take’ games: parent and infant exchange objects in a playful interaction and the parent often labels the name of the object so that the infant can easily grasp it. First words are acquired very slowly and are context-bound, meaning that there is direct, perceptual information about the link between the word and the object, and infants typically build a 10-word productive vocabulary by adding only one to three words a month^34,38^.

The decontextualization of words (the ability to use a word outside conventional routines) is considered ‘the hallmark of reference’^40^ as it indicates that children seem to discover that things have names and that “I can ask the name of an object if I don’t know it”. This *naming insight*^41^ signals an increase in vocabulary growth. At about 17 to 19 months of age, an infant’s vocabulary can expand at a rate of over five new words a week, a phenomenon termed ‘vocabulary spurt’^42^. By 18 months of age, English speaking infants usually reach a vocabulary of about 90 words; by 24 months, they may be able to produce 300 words^43–45^. As the rate of vocabulary increases, infants can refer to a wider range of concrete and abstract concepts. The naming explosion typically observed around 18 months of age has been hypothesized to be related to the new milestone of being able to categorize objects^39,46^. This impressive word learning is made possible by the sophisticated socio-cognitive and learning skills that allow infants to establish a reference for many types of words^47^. Even though many studies have focused on language acquisition and knowledge, no study has systematically addressed the emergence of abstractness—and proposed a distinction between reference and abstractness—in the acquisition of productive vocabulary of young children.

### Aims of the present study

Adopting a grounded view on abstract concepts, i.e., the Words as Social Tools^14^, we aim at documenting the development and trajectory of the acquisition of abstractness in word production during the developmental transition in language from 11 to 24-months of age in the context of frames for mother-infant toy play and adopting a microgenetic research design. This key transition is of special relevance since one-year-olds are active word learners, with a mean receptive vocabulary of around 80 words^37^ and with at least some words denoting abstract concepts^31^. Still, they are not yet able to produce any words referring to abstract concepts. Observing this developmental window, we will investigate abstract and concrete concepts as they are conveyed and expressed by concrete and abstract words. Notably, here we focus on “abstractness,” which we distinguish from “abstraction”^14,15,48^; see also^49^. With “abstraction,” we refer to the process that leads to the emergence of hierarchically ordered categories. Category formation always involves abstraction, and abstraction is more substantial the more general a category is. Hence, the abstraction process is more pronounced when we abstract from the distinctive characteristics of animals to form the superordinate category of “animals” than when we abstract from the features of specific cats to form the category “cat.” Still, in both cases, the referent of these categories consists of single objects/entities. Although related to abstraction, abstractness differs from it and concerns the characteristics of concepts whose referents are not single, bounded objects and the process that leads us to go beyond what we perceive through our senses, as when we interpret the experience of running on the grass as indicating “freedom”.

Considering the challenge to code abstractness in infancy we propose to operationalize it by looking at instances in which children use words that go beyond the ‘here and now’ context and moment, including therefore labels that refer either to abstract ideas or events displaced in space or time (past or future). References to internal states (volitions, emotions, cognitions), relational words, verbs and pronouns are also included in our classification, as they can vary in their degree of distancing from the present moment and context, and therefore in the degree of abstractness. We propose a clear distinction between reference and abstractness. Reference, even reference for absent objects, is still a concrete cognitive task: objects have names and can potentially be pointed at. Abstractness requires a detachment from concreteness and context, and reflects a form of thought/mental processing that is more closely tied to pretend, imagination, etc., than to reference. Social interaction is important in establishing reference, as a child needs to follow the pointing gesture of another person in order to understand which object the person is referring to. To share an abstract meaning, a different and specific kind of social interaction and social cognition is necessary, one that implies ‘not knowing’ and going beyond the ‘here and now.’ This creates the conditions for abstract thinking, and will change and re-organize the way in which the child interacts with other communicative partners. As a preferential avenue for higher-order cognition and abstractness, it is therefore essential for researchers to pay attention to the social context in which abstract words are acquired.

Compared to previous studies, ours has an important novelty. We intend to investigate the emergence and use of types of words differing in abstractness level as they emerge in real time in language acquisition. Importantly, we will pursue this aim employing an ecological method, i.e., analyzing the word usage in an interactive mother–child situation, when parent and infant play with objects. To the best of our knowledge, this is the first study systematically addressing the acquisition of different kinds of words denoting abstractness in infancy with such an approach. The few papers investigating the acquisition of concrete and abstract words in infants rely on questionnaires submitted to parents^50^—a methodology that may have a subjective component, as parents may be biased when reporting about their children’s knowledge^51^ or focus on the acquisition of words denoting single concepts (e.g., quantity^52^). The choice to adopt an ecological, longitudinal and interactive method is in keeping with our theoretical stance, according to which linguistic and social interaction are critical for the acquisition of a vocabulary referring to abstract concepts^53,54^.

Importantly, not only will we test the emergence of abstract words as a whole, but we provide a fine-grained analysis of different kinds of words denoting different level of abstractness, in line with recent trends in the literature on abstract conceptual knowledge^25–28,55–58^.

An important issue we intend to test is whether the early development of words referring to abstract, relational concepts facilitates later vocabulary acquisition. Lewis and colleagues^50^, relying on indirect measures of children's productive vocabulary, recently found evidence that knowing general, superordinate words facilitates faster vocabulary growth. Having the same vocabulary size but more superordinate words, such as ‘color’, ‘animal’, and ‘move’ may predict faster learning of words associated with those categories, rather than having less general words in one own’s vocabulary, such as ‘yellow’, ‘fox’, and ‘run’. According to Gentner^59^, words for relational concepts have a membership based on common relational structure; they include terms for spatial relations, and natural numbers and concepts like ‘friends’, ‘enemies’, and terms for kinship that have role slots. These terms can be learned via analogical comparison, which renders the common structure more salient, thus promoting abstraction and transfer^60^. Following previous research^14,50,59^, we expect that once children will develop the kind of cognition that allows for abstractness to which we referred above, they will also begin to utter and use some abstract words, and in particular relational terms, these labels will work as tools for learning other abstract words and concepts. Abstract words will help children retain and reuse relational structures, promoting future relational and abstract encoding and overall vocabulary growth. So, we expect an acceleration in the rate of acquisition of vocabulary once children begin to produce and use abstract words in conversation with their parents.

## Method

A microgenetic research design with a multiple case study method and a combination of quantitative and qualitative analyses^3,7^ was used to investigate the emergence of abstractness in vocabulary acquisition in the context of parent-infant games with objects during the second year of life. The data collection was part of a larger study directed by Alan Fogel at Purdue University on the development of communicative behavior in infants^61^. All methods in the study were carried out in accordance with the Declaration of Helsinki. Observational protocols and parent Informed Consent documents were approved by the Institutional Review Board of Purdue University (USA). Informed consent of the parents was obtained after the nature of the procedures had been fully explained.

### Participants

Eight children, four boys (subjects 1, 2, 3, and 4) and four girls (subjects 5, 6, 7, and 8), were videotaped biweekly interacting with their mothers and a set of age-appropriate toys in a laboratory playroom. The observation started when infants were twelve months and continued until they were 24 months. All children were full-term and healthy at birth. The mothers came from middle-class families in a small midwestern community in the United States, and they were all older than 21 years. All mothers spoke English as their primary language. One mother and infant were African-American, and the others were Caucasian-American.

### Procedure

Each session took place in a laboratory playroom. The mother and infant sat on small chairs at two adjacent sides of a low table, and the toys provided were two telephones, a doll bath and a crib set, a tea party set, and a wooden puzzle. Toys were selected to elicit both symbolic and functional play. The mothers were not informed of any research interest in abstract words. They were told: ‘Play and talk to your baby as you would at home’. This instruction was given only on the first visit to the lab. Thereafter, the mother was left to develop her own style of interaction with the infant. Attrition and missed visits were kept to a minimum by regular telephone contact with the staff, bi-monthly newsletter for the parents, and subject payments at the end of every 6-month period. One dyad completed all 30 possible sessions. The others completed between 26 and 29 sessions.

### Coding

The present work was based on a longitudinal database made available by Alan Fogel and on the transcription and coding of child’s vocabulary production from previously published studies^62^. To the best of our knowledge, English concreteness norms are only available for adults and not for children^63^. So, we developed a coding scheme that allowed us to account for the fact that—as we discuss later— while a concept might be relatively concrete for an adult, it might be considered as more abstract by a child (but see Section1 in the Supplementary Materials for an exploratory analysis using available databases). All proto-words and words used by the children in a play toy session with their mother were transcribed and coded from the tapes and further classified—for the current study—in terms of (a) grammatical category, (b) conceptual aspect and (c) level of abstractness. Only words that were uttered spontaneously were considered, not those that were produced as an immediate imitation of mother’s previous utterances. Below we describe more in detail the coding scheme adopted for the present study.

- Grammatical category. For each word uttered, we reported its corresponding grammatical category. For example, “dolly” was coded as a noun, “this” as a pronoun, “pretty” as an adjective, “take” as a verb.
- Conceptual level. Following the classification given in a widely used parent-report instrument to measure early language acquisition, the MacArthur-Bates Communicative Development Inventory (CDI) “Words and Sentences”^43^, we assigned each word uttered by the child to one of the categories according to the CDI: Animals, Toys, Drink, Body parts, Furniture, Small household items, Outside things/places, common nouns, People, Games and routines, Action words, Time words, Descriptive words, Pronouns, Question words, Preposition and locations, Numerals and quantifiers, Classifiers.
- Level of abstractness. Following the previous classification of abstract terms^29,64^ and the developmental classification of decontextualized terms and of terms indexing social knowledge^65,66^ three levels of abstractness were proposed—(1) low: concrete; (2) intermediate: medium; and (3) high: abstract. We coded as concrete words referring to objects or people that were physically present, either manipulable or simply visible, in line with the idea that concrete concepts are highly imageable, score high in Body Object Interaction, and are strongly associated with a specific context^15,29,67,68^. We included into this classification greeting routines, because they are directly linked to the appearance of objects and people, and terms referring to colors, because directly linked with visual aspects. We coded words as having an intermediate level of abstractness when: they referred to objects and people physically absent (displaced in space from the here and now situation), complex communicative and action routines, evaluative words that refer to physical appearance, spatial, time and location terms, and labels for physiological inner states. We considered labels for locations as intermediate because they do not have a single, spatially bounded object as referent and coded spatial and time terms as intermediate in line with previous evidence showing that spatio-temporal elements are the most concrete among abstract concepts^29^. Labels for perceptual and physiological inner states were taken as intermediate, in line with the idea that bodily states, and at a higher level of abstractness emotions, represent the first concepts children acquire that do not have a concrete referent^69,70^. Finally, we coded as high in abstractness words that are considered as such according to the current literature^26^, as words referring to numbers, emotions, and mental states terms, together with verbs and pronouns, typically considered as more abstract than nouns^71–73^, and words for routines that are considered as abstract in recent studies^31^.

We report below the coding schema enriched by specific examples. Level 1: concrete (low) was assigned to all names referring to objects that were perceptually present and that the child could manipulate (e.g., ‘doll’, ‘puzzle’, ‘plate’, ‘spoon’), to visible parts of the body (e.g., ‘hand’), to words referring to the mother (e.g., ‘mommy’), to colors (e.g., ‘red’), and to routines for greetings (e.g., ‘hi’, ‘bye’). Level 2: medium (intermediate) was assigned to names referring to absent relatives (e.g., ‘daddy’, ‘brother’, ‘grandma’) and to parts of one’s body that were invisible (e.g., referring to one's ‘nose’ or ‘eyes’). We considered concepts referring to invisible or absent elements in the intermediate category because these concepts do have a spatially bounded, concrete referent, like concrete concepts, but children recognize the current absence of that referent.

Level 2 was also assigned to routines (‘thanks’, ‘sorry’, ‘fine’), to onomatopoeic sounds or protowords (such as ‘roar’ for tiger, ‘gnam-gnam’ for eating), to question words (e.g., ‘what?’, ‘where?’), to descriptive words (e.g., ‘pretty’, ‘better’, ‘nice’), to references to space and time (e.g., ‘this’, ‘that’, ‘night’, ‘here’), to location words (e.g., ‘back’, ‘there’, ‘down’, ‘inside’, ‘up’, ‘on’) and to perceptual and physiological internal states (e.g., ‘see’, ‘look’, ‘hot’). Level 3: abstract (high) was assigned to personal pronouns, numbers and quantifiers (e.g., ‘all’, ‘another’, ‘more’, ‘other’, ‘some’), routines for affirming and negating (e.g., ‘yes’, ‘no’), to verbs referring to actions and to the routines ‘all gone’ and ‘all done’ (see also^31^). Internal states referring to emotions (e.g.,‘love’, ‘like’), volitions (e.g., ‘want’, ‘need’), cognitions (e.g., ‘think’, ‘pretend’), and moral terms (e.g., ‘good’, ‘bad’) were also assigned to level 3.

In order to have a sufficient number of instances for the analysis of quantitative changes in vocabulary acquisition, we divided the developmental time of the observations in four age groups. Age group 1 comprises observations from 12 to 15 months; age group 2 refers to observations from 16 to 18 months; age group 3 from 19 to 21 months, and age group 4 from 22 to 24 months.

## Results

To give a detailed picture of the emergence of abstractness in vocabulary acquisition, we will first present a qualitative analysis of types of abstract words and the corresponding age of acquisition observed for each term acquired by each child. Second, we will turn to an analysis based on the overall frequencies (tokens) of the different categories of terms uttered by children at different ages. We will report descriptives of the occurrences of the different categories of concrete, medium in abstractness, and high in abstractness, to show which categories children mostly rely on during the developmental period examined. Finally, to further explore the relation between age groups and conceptual categories, we will present correspondence analyses based on the frequencies of use of the categories of concrete and abstract words occurring in all age groups.

### Age of acquisition in real time and composition of abstract vocabulary

In this section, we will focus on a qualitative analysis of types of abstract words used by children while playing with their mother and the corresponding age of acquisition. Appendix 1 (in Section 3 of the Supplementary Material) presents the composition of vocabulary high in level of abstractness for each child and the corresponding age of acquisition (in weeks) for each term. As can be seen, children begin to use abstract words around 15 months; the first types of abstract words children produce are ‘no’, ‘yes’, personal pronouns for self—either ‘I’ or ‘me’—and the words ‘alldone’ and ‘allgone’. Two children already start to refer to the numbers ‘one’ and ‘two’ at 15 months, the others later on. Around 20 months children begin to use action verbs such as ‘cook’, ‘drink’, ‘sing’, ‘round’, and their first references to internal states ‘love’, ‘happy’, ‘want’, often in pretend games.

These terms were used in spontaneous conversation with the mother during play with age-appropriate toys. Below we provide real examples of the exchanges occurring between child and mother. A child (77 weeks) says ‘Alldone’ while giving back a toy to mother, a child says ‘empty’ (86 weeks) while putting the lid on the top of a pot, a child says ‘round’ when he turns around a piece of a puzzle to make it fit, a child (98 weeks) says ‘turn’ when he turns the puzzle so that it spins around, a child (94 weeks) says ‘very good’ when he did fit the piece of a puzzle in the right spot, a child (97 weeks) says: ‘Mama take it’ while offering the doll to the mother, a child wants to put the blanket on the doll by herself (without mother help) and says ‘I do’ (97 weeks), a child offers an apple to the doll and says ‘Like that?’ (age 102 weeks), a child (104) says ‘more milk’ while mother is pretending to drink, as he wants her to pretend to drink more, a child (87 weeks) says ‘cry’ while holding a doll, in reply to the mother’s comment: ‘You better hold her nicely’, the mother than asks ‘is she crying?, and the girl responds ‘yes’. These excerpts show that the terms were used within a dialogical exchange, and were genuine and creative references to abstract aspects rather than contextualized occurrences.

Appendix 2 (in Section 3 of the Supplementary Material) reports the first ten words and their age of acquisition (in weeks), and size of overall productive vocabulary at 24 months for each child. The first types of concrete words children use are: ‘mama’, ‘baby’ (for the doll), ‘ball’, ‘bottle’, ‘cup’, mostly referring to toys they are playing with, or they want to play with. They also use simple forms of greetings, such as ‘bye’ and ‘hi’, that we classified as concrete.

There was a large interindividual difference in vocabulary sizes in our sample, well documented in the literature for early stages in language acquisition^44^. However, when we look at the relation between the size of abstract vocabulary and overall productive vocabulary that the children reached by 24 months, we notice an interesting progression. The magnitude of abstract vocabulary was positively correlated with the overall magnitude of vocabulary size across children, *r*(6) = 0.94, *p* > 0.001. In fact, children reaching larger abstract vocabulary at 24 months tend to have larger overall vocabulary as well, and the group of children with a smaller abstract vocabulary at 24 months have smaller overall vocabulary as well. Remarkably, this is true independent of the child’s gender. We can more easily observe this progression by ordering children on the basis of their size of abstract vocabulary, as shown in Appendix 3 (in Section 3 of the Supplementary Material). This insight seems to support the hypothesis that children acquiring an earlier ‘kit’ of abstract, relational concepts tend to be faster vocabulary learners by the age of 24 months. Alternatively, and in line with views according to which language plays a pivotal role for abstract concepts representation and use, abstract concepts emerge only when children possess a sufficiently broad vocabulary^74^. Further research is needed to untwine these two alternative hypotheses.

### Patterns in production of concrete, medium and abstract terms

**Figure 1:**
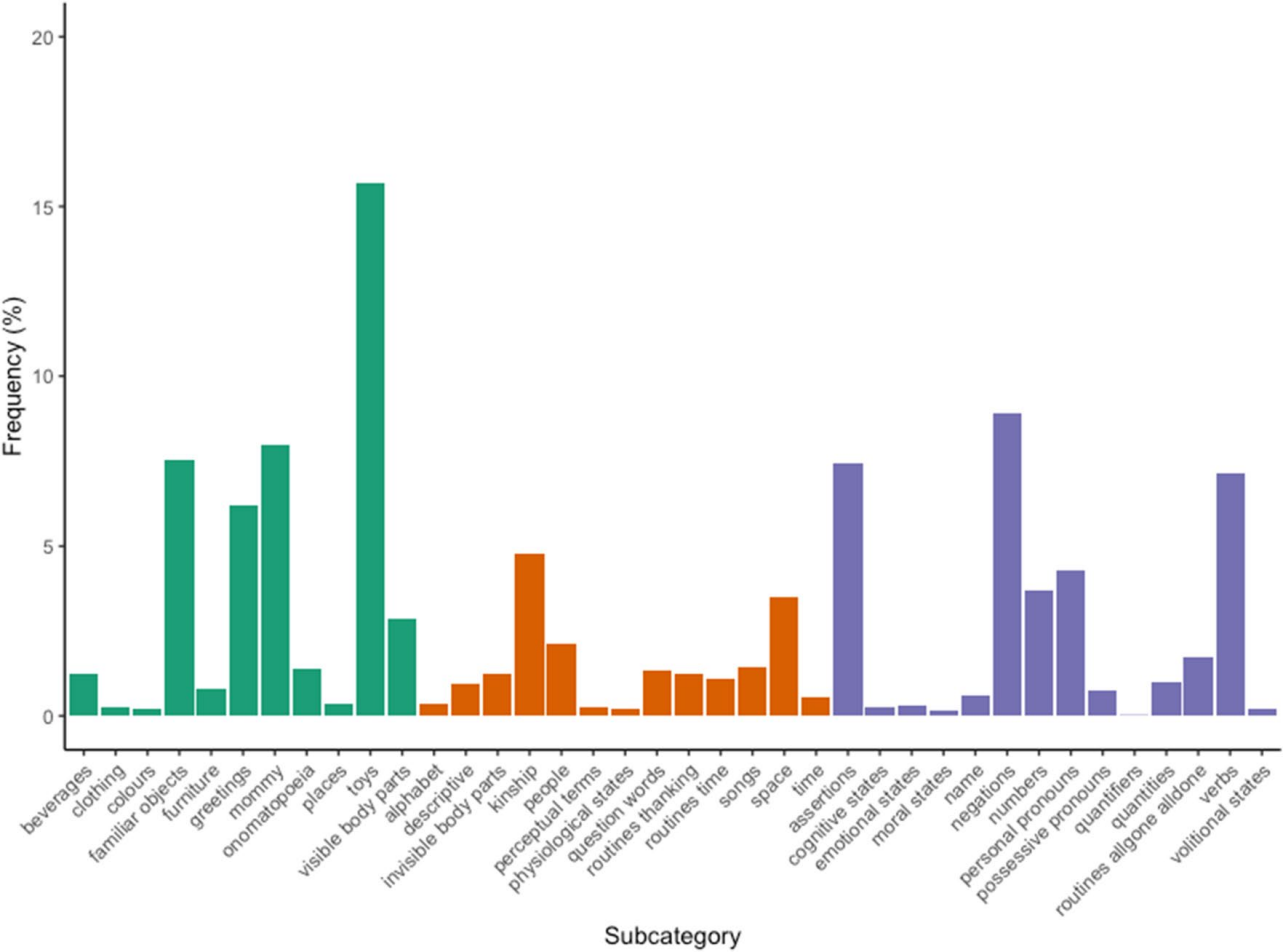
Percentage of words produced by children across age groups for all subcategories. Green bars represent concrete subcategories, orange bars represent subcategories with an intermediate level of abstractness, blue bars represent abstract subcategories.

To explore the pattern of all kinds of words produced by children at different ages, we present percentages of words produced for each subcategory by children across all sessions. Subcategories are in different colors depending on their level of abstractness. Figure 1 shows the overall percentage of words produced for each subcategory by children across all sessions.

Concrete (green): names for toys (e.g. ‘doll’, ‘puzzle’, ‘plate’), for visible body parts (e.g. ‘hand’), names referring to the mother (present), names for vehicles, beverages, clothing, furniture, familiar objects, places, routines for greeting (‘hi’, ‘bye’), and colours. Figure 1 shows the overall percentage of words produced for each subcategory by children across all sessions.

Medium (orange): kinship (e.g. ‘daddy’, ‘brother’), names of people, own’s body parts that cannot be directly seen (e.g. ‘nose’ or ‘eyes’), routines such as ‘thank’, onomatopoeic sounds (such as ‘roar’ for tiger), descriptive words, question words (where, what), references to space and time (e.g. ‘night’, ‘here’), and to perceptual and physiological internal states (e.g. ‘see’, ‘’hot’), alphabet and songs.

Abstract (blue): personal and possessive pronouns, child’s own name, numbers, quantifiers, routines for affirming and negating, verbs for actions and verbs for routines ‘all gone,’ ‘all done,’ internal states referring to emotions, desires, volitions cognitions and moral states.

Across all age groups, the more frequent concrete concepts are toys, familiar objects, references to their mommy, and greeting routines. The more frequent middle concepts are kinship and spatial terms. Finally, the more frequent abstract concepts are assertions, negations, verbs, and personal and possessive pronouns. Table S2 in Section 2 of the Supplementary Materials presents frequencies and percentages of production of each subcategory across sessions.

To tackle the developmental pattern of abstractness, we now consider the production of children, breaking it down for each age group (see §Coding).

**Figure 2:**
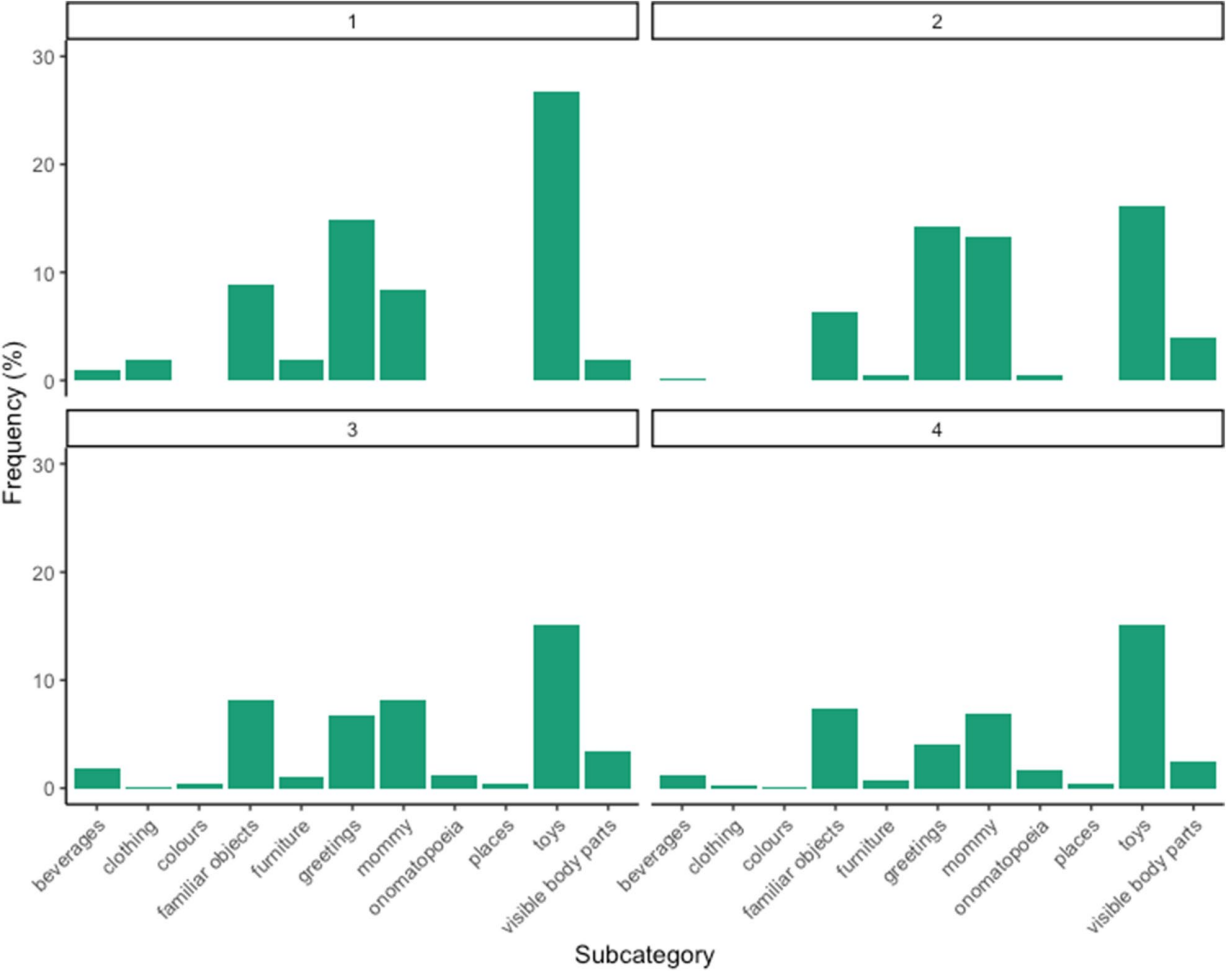
Percentage of words produced by children of age group 1 (12–15 months), age group 2 (16–18 months), age group 3 (19–21 months), and age group 4 (22–24 months) for concrete subcategories.

**Figure 3:**
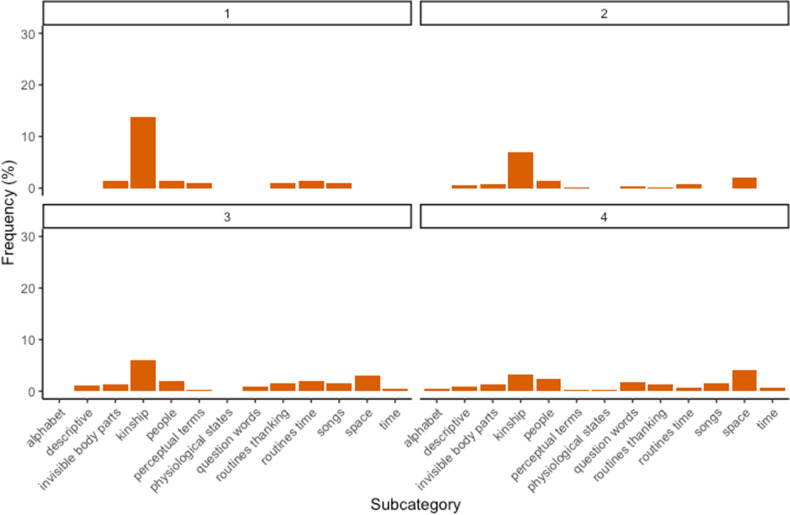
Percentage of words produced by children of age group 1 (12–15 months), age group 2 (16–18 months), age group 3 (19–21 months) and age group 4 (22–24 months) for subcategories with medium level of abstractness.

**Figure 4:**
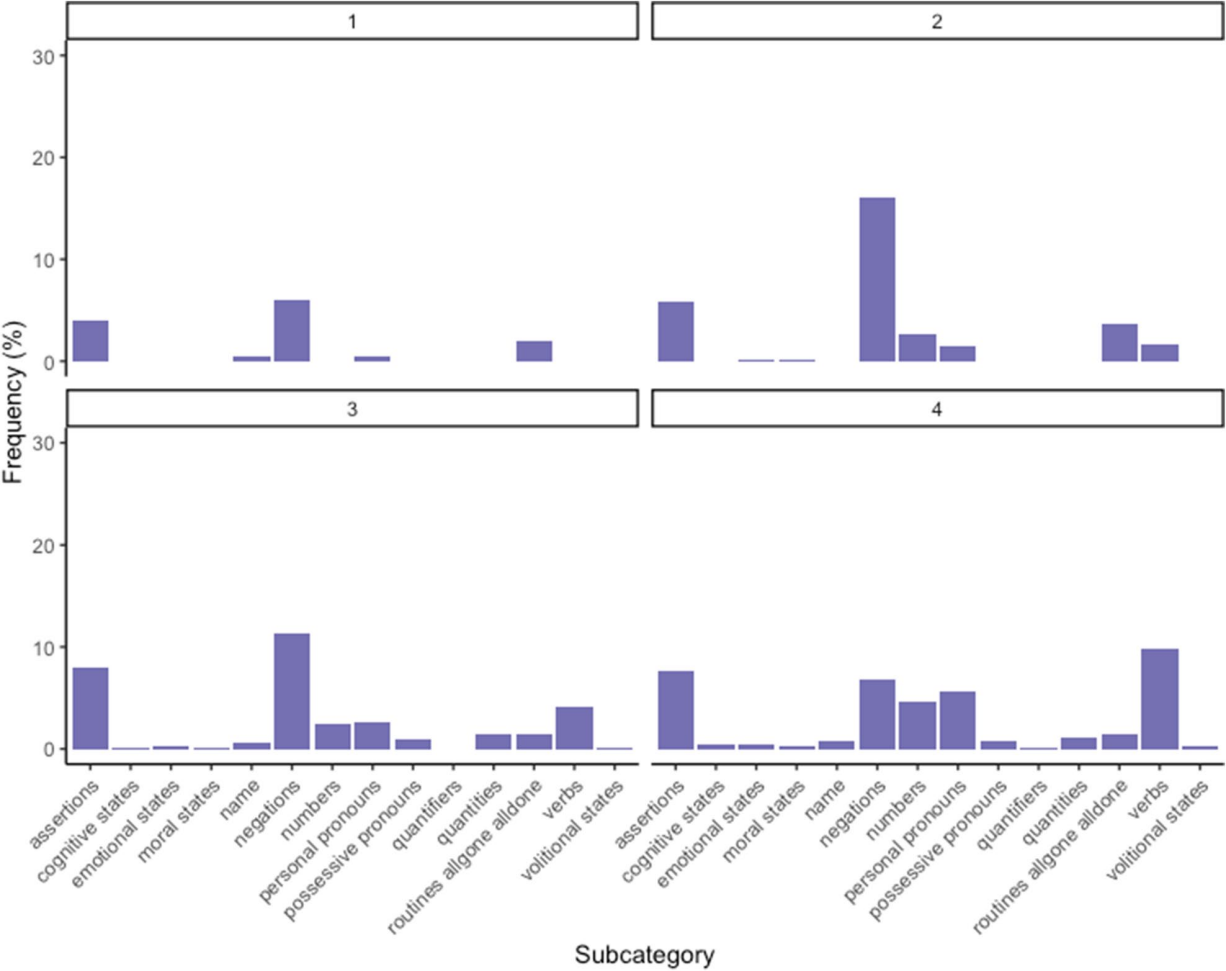
Percentage of words produced by children of age group 1 (12–15 months), age group 2 (16–18 months), age group 3 (19–21 months) and age group 4 (22–24 months) for subcategories with high level of abstractness.

Figures 2, 3 and 4 present the percentage of words produced for each subcategory in each group of age, respectively for concrete, medium and high level of abstractness.

Considering the subcategories for each age group (Figs. 2, 3 and 4), we can notice slight variations across ages. Within concrete concepts, the subcategories of greetings and mommy slightly decrease. Within middle concepts, there is a slight decrease in the subcategory of kinship. More interestingly, we can notice a progression in abstract concepts: negations, numbers, and personal pronouns emerge in the second age group, while assertions increase progressively with age. Finally, quantities appear in the last two age groups, while routines allgone-alldone are mainly produced by younger children.

### Relation between categories and age

**Figure 5:**
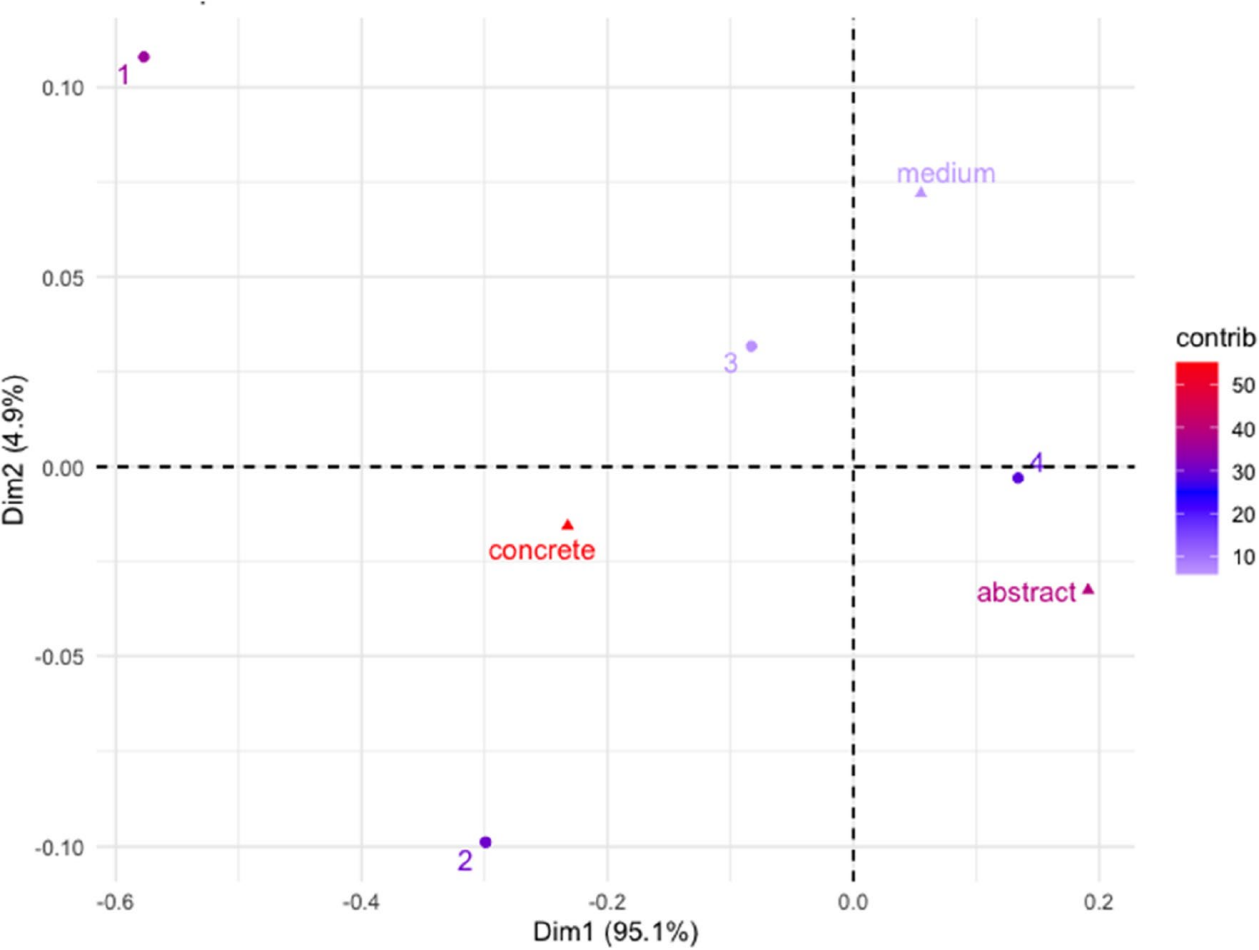
Symmetric plot of Dimensions 1 and 2, with points distribution and their contribution on dimensions. Rows are represented by circles and columns by triangles. The distance between row points or column points represents their relation. Closer points are more similar to each other than more distant points.

To further address the relation between age group and category, we conducted a correspondence analysis, implemented through the “FactoMineR” and “factoextra” R’s packages^75,76^. Correspondence Analysis is a data reduction technique allowing to extract the main dimensions along which information is grouped^77^. The input for the following correspondence analysis is a matrix composed of age groups (rows) and the sum of occurrences produced by each age group for each category (columns). We found a statistically significant dependency between rows and columns, ${\rm X}^{2}$(6) = 199.72, *p* < 0.001. The first two dimensions explained 100% of the variance, with Dimension 1 explaining most of the variance (95.05%), followed by Dimension 2 (4.94%). The results can be seen in Fig. 5.

We discuss only points that weigh more than 10% of the variance for each dimension.

Dimension 1 captures the opposition between abstractness and concreteness, featuring a distinction between concrete and abstract concepts, the first characterizing the production of younger children and the second of the older age group. Thus, on Dimension 1 (which explains most of the variance), we find a strong opposition between age group 1 (12–15 months, 35.81% of variance) and age group 4 (22–24 months, 30%), while on Dimension 2, age group 2 (16–18 months, 60.21%) is opposed to age group 3 (19–21 months, 15.39%).

Dimension 1 features the traditional opposition between abstract (39.9%) and concrete (57.89%) concepts, while on Dimension 2, we find medium concepts (72.62%) opposed to abstract concepts (22.28%).

Results from the correspondence analysis suggest that children in the first age group (12–15 months) produced more concrete concepts, whereas they produced more abstract concepts as they grew older (age group 4, 22–24 months). As for the middle-aged groups, we found children in the second age group (16–18 months) produced more abstract concepts, while children in the third age group (19–21 months) mainly produced concepts at an intermediate level of abstractness. The last result might not seem intuitive, but it accounts for a very small percentage of the variance (4%), and it can be considered marginal. It can, however, remind us that the process from concreteness to abstractness is not straightforward, but there might be fluctuations.

Table S3 in Section 2 of the Supplementary material presents descriptives of production of abstract, concrete, and medium terms produced in each age group.

A qualitative inspection of the data shows that, on average, children tended to produce more concrete terms in all age groups except from age group 4—in which the production of abstract terms increased, exceeding the production of both concrete and medium terms.

**Figure 6:**
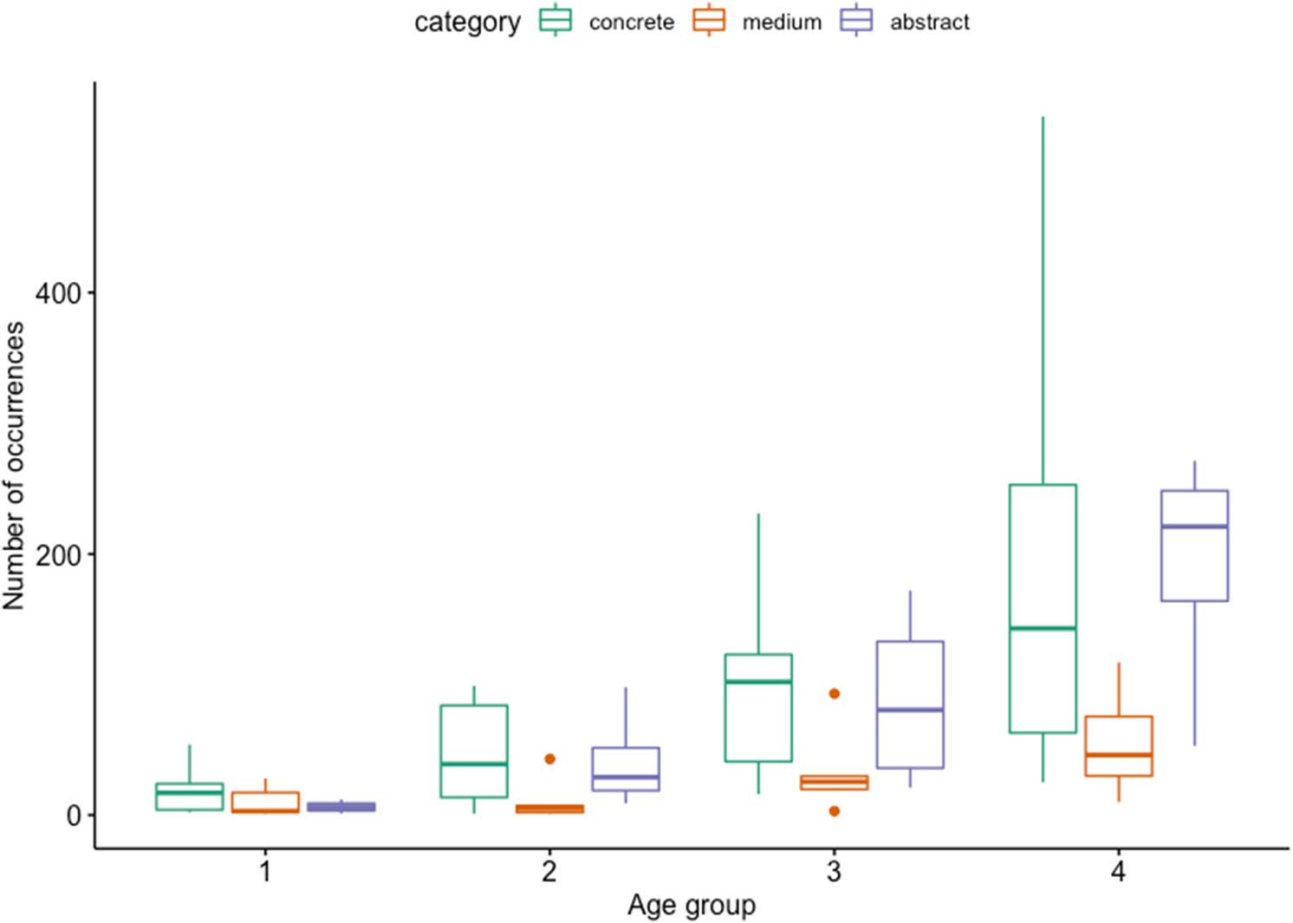
Distribution of occurrences of abstract, concrete, and medium words for each age stage. Bars represent medians.

Figure 6 shows the distribution of occurrences of concrete, medium, and abstract words for each age stage.

Despite large interindividual differences, there is a growing trend for both concrete words and for words with medium and high levels of abstractness across sessions. Concrete words are used proportionally more than abstract words.

*Relationship between age groups and subcategories.* To further explore the pattern of our data, we performed another correspondence analysis using a matrix composed of age groups (rows) and the sum of occurrences produced by each age group for each subcategory (columns). We found a statistically significant dependency between rows and columns, ${\rm X}^{2}$(48) = 378.69, *p* < 0.001. The first two dimensions explained 87% of the variance, with Dimension 1 explaining 56.31% of the variance, followed by Dimension 2 explaining 31.45% of the variance, and Dimension 3 explaining 12.2% of the variance.

We discuss only points that weigh more than 10% of variance.

On Dimension 1 age group 2 (16–18 months, 40.60%) and age group 1 (12–15 months, 22.10%) are opposed to age group 4 (22–24 months, 33.69%), while on Dimension 2 age group 1 (12–15 months, 72.71%) is opposed to age group 2 (16–18 months, 22.83%). On Dimension 3 we found a strong opposition between group 2 (16–18 months, 23.87%) and group 3 (19–21 months, 65.40%).

**Figure 7:**
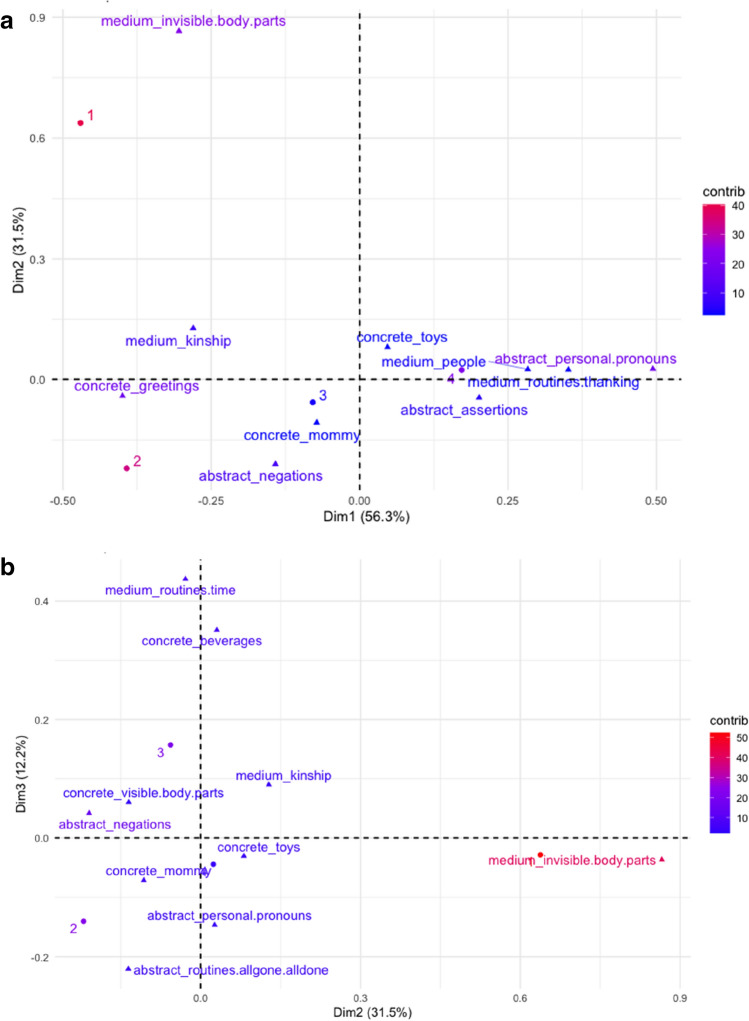
(**a**) Symmetric plot of Dimensions 1 and 2, with points distribution and their contribution on dimensions. Only the top 10 contributing columns (subcategories of concepts) are shown. Rows are represented by circles and columns by triangles. The distance between row points or column points represents their relation. Closer points are more similar to each other than more distant points. (**b**) Symmetric plot of Dimensions 2 and 3, with points distribution and their contribution on dimensions. Only the top 10 contributing columns (subcategories of concepts) are shown. Rows are represented by circles and columns by triangles. The distance between row points or column points represents their relation. Closer points are more similar to each other than more distant points.

On Dimension 1, we found no point contributed more than 10% to the variance. However, it might be worth mentioning three categories that contributed more than 8% to the variance: abstract routines allgone-alldone (9.15%) opposed to concrete toys (9.49%) and abstract assertions (8.27%). On Dimension 2, we found an opposition between medium invisible body parts (57.25%) and abstract negation (19.28%), while on Dimension 3 we found concrete beverages (19.14%) together with medium routines-time concepts (26.01%) are opposed to abstract routines allgone-alldone (10.48%) and abstract personal pronouns (11.66%). Figure 7a shows the distribution of points on the bidimensional space constituted by Dimension 1 and 2, and Fig. 7b by Dimension 2 and 3.

The correspondence analysis results indicate that children in the first age group (12–15 months) produced more words referring to concepts with a medium level of abstractness, labels for body parts. In contrast, children in the second group of age (16–18 months) produced mainly abstract concepts referring to routines-negation. They also suggest that children in the first (12–15 months) and second (16–18 months) group of age produced the first words for abstract concepts, i.e., abstract routines allgone-alldone. Children in the second group of age (16–18 months) produced mostly words for abstract routines such as allgone-alldone and abstract personal pronouns; children in the third group of age (19–21 months) used mainly medium routines-time related terms and concrete terms referring to beverages; they also produced labels for concrete toys and abstract-assertions.

The results of this analysis give us important information as to the development of a vocabulary for different kinds of abstract concepts. We first find a clear opposition between younger and older infants (Dimension 1, group 1, 12–15 months and 2 versus group 2, 16–18 months). Even if no point weighs more than 10%, an analysis of points contributing above 8% suggests that children of the first two age groups already produce the first abstract words (routines allgone-alldone), but that older children produce more sophisticated words for abstract concepts, i.e., abstract assertions, together with concrete toys. An increase in abstractness seems to occur early on: in the second dimension, we find that, whereas children of age group 1 (12–15 months) produce more labels for invisible body parts (medium), children of age group 2 (16–18 months) produce more abstract negations. Importantly, there might be shifts and fluctuations, especially between age groups 2 (16–18 months) and 3 (19–21 months), as revealed by Dimension 3: children of age group 2 (16–18 months) produce more abstract personal pronouns and routines alldone-allgone, children of age group 3 (19–21 months) more concrete beverages and routines-time (medium).

## Discussion

The results of the study are quite clear. They indicate that children during the course of the second year of life use progressively more abstract words when interacting with their mothers in a toy play context. The major shift seems to occur between the first and last phases, i.e., from 12–15 months to 22–24 months, but the qualitative data indicate an incremental growth of terms to denote abstract concepts. Our results confirm that the acquisition of words referring to abstract concepts occurs later compared to that of words referring to concrete ones. This evidence complements that of language production in children and adults. For example, Brown (1957), as reported in Schwanenflugel^78^, suggests that 75% of the words most frequently produced by school‐age children (first to eighth grade) were concrete, while only 28% of the most used words by adults were concrete^69^.

Previous work on the acquisition of concrete vs. abstract terms in productive vocabulary has relied on parental reports identifying the age of children’s first usage of specific terms^37,50^. Parental reports have the advantage to acquire quick data collection on large samples, but at the expense of reliability. When asked to check from a list of words those ever used by their child, parents may overestimate or underestimate their children’s productive vocabulary and tend to respond on the basis of their memories, perceptions, and beliefs, as they do not have specific training on language development; they may also respond on the basis of what they think is more acceptable or expected, the well-known ‘social desirability’ effect. Laboratory and naturalistic observations, upon which our work relied, require transcription of speech and are labour intensive, but have the undeniable advantage of ecological validity and are considered direct, objective and reliable^51,79^. Our study is the first one, to our knowledge, focusing on the real time age of acquisition for abstract terms based on direct longitudinal observations (rather than on parental reports) of the child in interaction with the mother during the second year of life. Parent-infant play can be considered a privileged situation that facilitates symbolic thinking and early language acquisition. The microgenetic design adopted, using multiple case studies and intensive observations across a key developmental transition, enabled us to document the composition and the age of acquisition of abstract terms in real-time for eight English-speaking children. Despite ample interindividual differences observed in our sample in abstract and overall vocabulary sizes, we found a relationship between the number of abstract words and overall vocabulary size for children, giving initial support to the idea that abstract vocabulary can be seen as a kit or a tool for improving the elaboration of abstract cognitive skills and the resulting production of abstract words in spoken language. Our results have several implications bridging research on abstract conceptual knowledge and developmental research. In the following, we will briefly summarize the main findings of our study.

First, we provide evidence of a significant shift from terms denoting concrete to abstract concepts between one and two-year-olds. Notably, terms denoting abstract concepts produced at this age are not like “fantasy” or “justice”. We offered a framework to operationalize and classify different kinds of abstract terms very early in development, and proposed that abstractness requires a detachment from concretness and context and a kind of social interaction and cognition that implies ‘not knowing’ and going beyond the here and now.

Second, and crucially, we presented a detailed analysis of the pattern of categories that progressively emerge as more relevant with age. In accord with recent developments in the literature on abstractness, we show that categories of words used to refer to abstract concepts are not a unitary whole but are rather composed of different kinds^25,27,58,80^. Within terms denoting abstract concepts, abstract routines alldone-allgone are present early, and characterize mostly the first age group (12–15 months); abstract negations and abstract personal pronouns emerge in the second age group (16–18 months), whereas the more complex abstract assertions emerge later on. Interestingly, labels for numbers appear in the second age group and quantities in the third (19–21 months). This result supports the idea that labels for numbers might be less abstract than labels for other abstract concepts, like mental states ones^58,81^.

Importantly, the analysis we performed on the emergence of the different subcategories of terms denoting abstract concepts can also contribute to very recent literature on abstract concepts in adults. It confirms that abstract concepts cannot be considered a unitary whole nor a dichotomy; they can be seen as a continuum’s extremes or, even better, as points in a multidimensional space defined by various dimensions, such as abstractness, imageability, age and modality of acquisition and that it is crucial to investigate fine-grained differences^26,27,55,58^.

Third, the qualitative analysis we performed allowed us to understand in detail the progression of abstract words acquisition in specific domains. Among the categories of abstract words analyzed in our work, two categories deserve special attention in our view, as they seem especially difficult to acquire: verbs and terms denoting mental states, which we will discuss next.

In relation to verbs, it is well established that verbs are acquired later than nouns in different languages and cultures—even in ‘verb-friendly’ languages such as Korean and Mandarin^37,82–84^. It has been proposed that nouns are easier to learn than verbs because they refer to perceptually distinct units that are stable and consistent across time and context^85^. Nouns rely on the basic understanding of object permanence and of the physical properties of objects that are well established in the first year of life^47,86^. By contrast, to learn a verb, children have to determine which aspect of an ongoing event is being referred to, so they have to understand something of the intentional nature of actions^47,87^—an abstraction—and these abilities develop toward the end of the second year. Golinkoff & Hirsh-Pasek^88^ propose that toddlers must coordinate multiple cues (perceptual, intentional, grammatical) to learn verbs, the gateway to grammar. They propose an interesting continuum representing the fact that verbs that rely on perceptual cues will be learned before verbs that label perceptually less accessible referents. According to their model attention to perceptual cues for verb meaning precedes children’s attention to intentional and grammatical cues, that would not be in the service of word learning until 24 months of age.

Furthermore, verbs are more complex than nouns since they involve a relation between at least one agent and some activity, and action categories are not as well defined as object categories^47,85,89^. Consistently, in our data, all children begin to use verbs only after 18 months. The first verbs uttered are ‘push’, ‘drink’, ‘hang up’, ‘done’, ‘stir’, and ‘don’t’, and by the end of the second year they can rely on a wide range of verbs including ‘play’, ‘tickle’, ‘call’, ‘make’, ‘count’, ‘work’, ‘sleep’, ‘go’, ‘wait’, ‘eat’ and ‘sing’.

In relation to mental states, several authors have documented that language for labelling states or processes occurring in the mind emerges late in the second year of life and grows in the third, as children’s conversations gradually come to carry explicit reference to shared inner experiences^90–92^. Internal state language is highly predictive of later Theory of Mind, as measured by the false belief task^93^. In our work, we classified these terms as abstract, as they refer to internal experiences that are not visible and that can only be inferred. The vocabulary for mental states collected from our sample is large and includes terms such as ‘pretend’, want’, ‘love’, ‘happy’, ‘feel’, ‘know’, ‘like’ and ‘worry’, showing that by 24 months children indeed have begun to reflect on themselves and on other’s behaviors in conversations with a parent in a somewhat abstract way.

Finally, our findings suggest a reason why learning progressively more abstract concepts might be crucial. In line with recent views, our results suggest that language, and particularly abstract language, promotes cognition. Specifically, we found that the frequency of the produced abstract terms corresponds to the vocabulary size—the larger the vocabulary size, the larger the number of produced abstract concepts. This finding might be interpreted in two different ways, both suggesting an important relationship between abstract concepts and language. First, we could say that children need to master many words to acquire hard words like abstract ones^74^. Alternatively, we could say that children who developed early abstract, relational concepts later have a wider vocabulary^50^. In a similar perspective, Lewis, Colunga & Lupyan^50^, recently demonstrated that knowing general words facilitates faster vocabulary growth. Why should abstract terms facilitate language learning—and possibly higher-order cognition? Gentner^59^ has proposed that there is a special class of terms referring to relational concepts that are important and difficult to acquire, but that represent a cognitive ‘tool kit’ and a preferential avenue for higher-order cognition and abstractness. Relational words are slow to be acquired, but some of these words, such as terms for spatial relations (e.g. ‘in’, ‘out’) and ordinal numbers, are acquired early and, once acquired, are in constant use. Our results add to these studies a detailed and fine-grained documentation of the specific abstract terms used in conversation with parents by typical English-speaking children.

Despite its ecological and longitudinal approach, our study is not without limitations. The microgenetic design adopted, using multiple case studies and intensive observations across a key developmental transition, has the disadvantage to allow data collection for only small numbers of participants. The conclusions gained from our effort may be tested in larger samples of children speaking different languages and belonging to more diverse socio-cultural backgrounds, in order to establish typical versus delayed trajectories of the acquisition of abstract vocabulary. Moreover, investigating factors that may underlie typical versus delayed acquisition of abstract vocabulary may in the future be relevant and useful in designing interventions for children with atypical language development. On the other hand, our focus on productive language acquisition constrained our work and our conclusions as it cannot be excluded that infants younger than one year may possess some abstract concepts even before having learned any words that refer to these concepts.

To conclude, our study provides insight into young children's progress in acquisition of abstract terms, combining qualitative examples with a quantitative analysis. We focused on how infants progressively acquire a detailed vocabulary to refer to abstract concepts with an ecological method, analyzing word usage in an interactive mother–child play situation. It documents when children start to use a wide range of abstract terms for the first time. Our results show that children acquire terms denoting abstract concepts later than terms denoting concrete ones, but not in a homogeneous manner since different sub kinds of abstract concepts emerge at different ages. It also suggests the exciting possibility that abstract concepts might prepare children to acquire more words and new sophisticated thinking abilities. Further research is needed to deepen this critical topic.

## Supplementary Information

Supplementary Information.

## Data Availability

The videos were donated by Alan Fogel to the Strong National Museum of Play www.museumofplay.org/ , which keeps an archive of the materials that can be used by any researcher. The datasets analysed during the current study are available on the open platform OSF at https://osf.io/jc9np/.
