# Supplementary material for: Abstractness emerges progressively over the second year of life
Source: Sci Rep. 2022 Dec 3;12:20940. doi: 10.1038/s41598-022-25426-5 (PMC9719541; doi:10.1038/s41598-022-25426-5)
Supplement: Supplementary file 1 — Supplementary Information. [file 41598_2022_25426_MOESM1_ESM.docx]

**Section 1: The emergence of abstractness and its relation with existing databases**

Here we explore whether the trend we observed in our data relying on abstractness~concreteness categories we created specifically for children holds when using established normative databases. In order to do so, we retrieved as many words as possible from Brysbaert et al.’s (2014) concreteness database among those produced by the children in our dataset. Before turning to the description of the comparison, two specifications are necessary. First, these exploratory analyses are only possible for words that we coded as either abstract or concrete, so the following does not apply to words coded as having “medium” levels of abstractness. Second, it has to be pointed out that Brysbaert et al.’s database was built by collecting ratings from adults, not from children. So, it might be possible that while a concept is generally considered to be concrete by an adult speaker, the same concept might have a different abstractness level for a child (see our coding scheme).

Out of 115 unique (abstract and concrete) words produced by our children, we found 99 words in the concreteness database. So, we refer to this subset of words for the following analyses. Overall, concreteness scores were comparable across age groups (age group 1 Concreteness *M*= 4.14; *SD* = 1.05; age group 2 Concreteness *M*= 4.41; *SD* = 0.94; age group 3 Concreteness *M*= 4.41; *SD* = 0.86; age group 4 Concreteness *M*= 4.35; *SD* = 0.96), (see Figure S1).


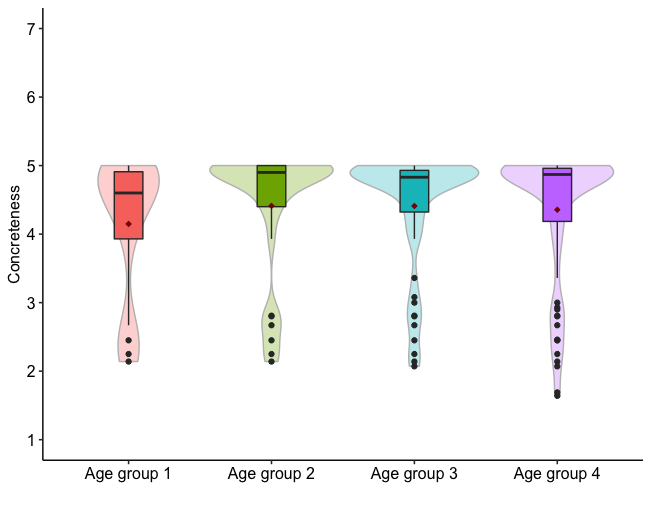


**Figure S1**

Average concreteness scores and their distribution retrieved from Brysbaert et al.’s (2014) database for all the matching words (N= 99) produced by our sample of children. Red squares represent means, black bars represent medians, and black dots represent extreme datapoints.

Next, we distinguished words according to the standard concreteness threshold of 3.5 to see how many abstract and how many concrete words we would have in the dataset in keeping with Brysbaert et al.’s database. Words with a concreteness score equal or below 3.5 in the concreteness database are classified as abstract, whereas words with a concreteness score above 3.5 are classified as concrete.

In keeping with that, we found 17 abstract and 82 concrete words in our dataset. Across all age groups, abstract and concrete words matching those in the concreteness database were similarly distributed. Specifically, 23.53% of the total sample of words (N= 17) retrieved in the database and produced by children in age group 1 were abstract, whereas 76.47% were concrete. In age group 2, 18.18% of the total (N= 33) of words were abstract, and 81.82% of words were concrete. Similarly, in age group 3 17.24% of the total (N= 58) were abstract words, while 82.76% were concrete words. Finally, among the total of words retrieved from age group 4 in the database (N= 78), 19.23% were abstract while 80.77% were concrete.

Table S1 presents the frequency of production of abstract and concrete words in our sample based on this distinction

**Table S1**

Frequency of production of abstract and concrete words classified according to their concreteness scores in Brysbaert et al.’s (2014) database.

| **Age group** | **Category** | **Frequency** | **Unique words** |
| --- | --- | --- | --- |
| 1 | abstract | 49 | 4 |
| 1 | concrete | 78 | 13 |
| 2 | abstract | 207 | 6 |
| 2 | concrete | 214 | 27 |
| 3 | abstract | 392 | 10 |
| 3 | concrete | 444 | 48 |
| 4 | abstract | 608 | 15 |
| 4 | concrete | 905 | 63 |

The overall trend of the distribution of abstract and concrete words across age groups seems to recapitulate the pattern we found in our main analyses—even though with slight variations. In summary, abstractness seems to emerge gradually across age groups (see Figure S2). Hence, we can notice that the main results of the qualitative and quantitative data do not consistently diverge. The differences occurring between the two databases might be explained by the fact that Brysbaert et al’s (2014) ratings are collected from adults, while our analysis concerns the production of children. A further reason that explains the difference lies in the fact that words in Brysbaert et al. (2014) were presented and evaluated against the entire range of word concreteness/abstractness. As a result, the words acquired early in life were likely to have been perceived as relatively more concrete, and the subtle change in abstractness might have been overshadowed. The current study focused on these early acquired words. When zooming in, we noticed the upward trend in word abstractness.


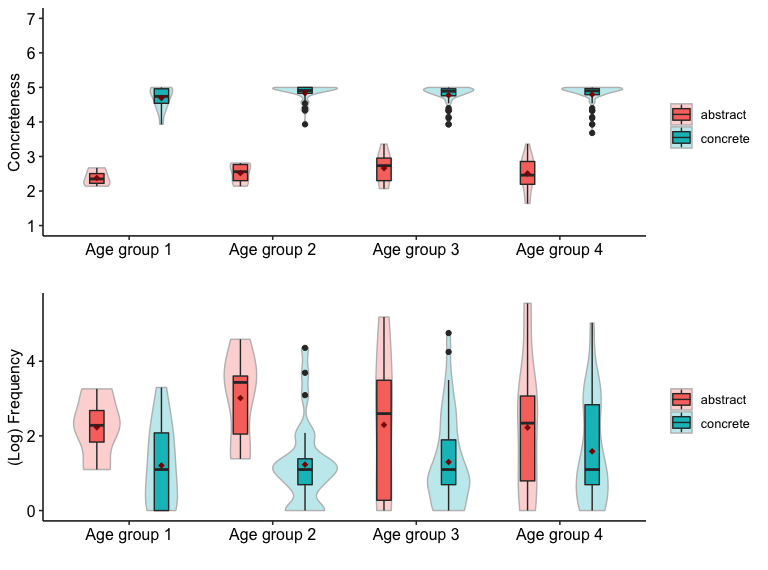


**Figure S2**

Concreteness scores retrieved from Brysbaert et al.’s (2014) database and (log) frequency of abstract and concrete words produced in each age group matching those in the concreteness database (N= 99). Means are represented with red squares, black bars represent medians, and black dots represent extreme datapoints.

**Section 2: Table S2 and Table S3**

**Table S2**

Frequencies and percentages of production of each subcategory across sessions


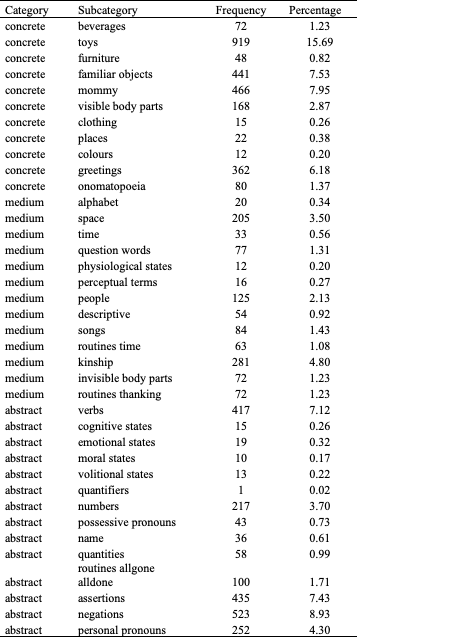


**Table S3**

Means, standard deviations, and raw frequency of abstract, concrete, and medium terms produced by children in each age group (1) months 12-15; 2) 16-18; 3) 19-21; 4) 22-24.)

| Age group | Abstractness | *M* | *SD* | Frequency |
| --- | --- | --- | --- | --- |
| 1 | abstract | 3.13 | 3.31 | 25 |
| 1 | concrete | 15.88 | 15.50 | 127 |
| 1 | medium | 6.50 | 8.75 | 52 |
| 2 | abstract | 22.13 | 14.69 | 177 |
| 2 | concrete | 39.75 | 38.85 | 318 |
| 2 | medium | 14.25 | 15.54 | 114 |
| 3 | abstract | 63.50 | 43.32 | 508 |
| 3 | concrete | 76.25 | 55.27 | 610 |
| 3 | medium | 48.88 | 29.43 | 391 |
| 4 | abstract | 171.13 | 126.89 | 1369 |
| 4 | concrete | 122.50 | 59.87 | 980 |
| 4 | medium | 103.38 | 51.01 | 827 |

**Section 3: Appendixes**

**Appendix 1**

Types and age of acquisition (in weeks) of words with high level of abstractness for each child

Boy 1

| Age in weeks | First emergence of the word |
| --- | --- |
| 67 | no |
| 77 | alldone |
|  | yes |
|  | two |
| 78 | push |
|  | my |
| 86 | pretend |
|  | more |
|  | empty |
|  | I |
|  | round |
|  | play |
|  | her |
|  | hang up |
| 90 | mine |
|  | go |
|  | three |
|  | happy |
| 92 | one |
|  | two |
| 94 | fall down |
|  | find |
|  | five |
|  | get |
| 96 | allgone |
|  | allright |
|  | sit |
|  | turn |
|  | tickle |
|  | play |
|  | want |
|  | more |
|  | child’s own name |
|  | outside |
| 98 | drop |
| 102 | cut |
| 104 | dump |
|  | call |

Boy 2

| Age in weeks | First emergence of the word |
| --- | --- |
| 63 | yes |
| 70 | no |
| 77 | drink |
| 83 | two |
|  | rock |
|  | ask |
|  | you |
| 85 | I |
|  | one |
| 87 | two |
|  | it |
|  | make |
|  | nine |
|  | research |
| 89 | fit |
| 91 | count |
|  | three |
|  | four |
| 93 | he |
|  | hang up |
|  | five |
|  | go |
|  | ten |
| 95 | fine |
|  | eight |
|  | nine |
|  | seven |
| 97 | sleep |
|  | round |
|  | me |
|  | ring |
|  | good |
| 102 | do |
|  | work |
|  | ring |
|  | fell down |
|  | do |

Boy 3

| Age in weeks | First emergence of the word |
| --- | --- |
| 48 | no |
|  | alldone |
| 66 | yes |
| 68 | allgone |
| 76 | go |
| 82 | hang up |
|  | brush |
|  | I |
|  | want |
| 90 | mine |
|  | me |
|  | pour |
| 104 | two |

Boy 4

| Age in weeks | First emergence of the word |
| --- | --- |
| 64 | no |
| 65 | yes |
| 73 | done |
| 76 | you |
| 78 | I |
| 81 | go |
|  | sit |
| 83 | Don’t |
| 84 | Own’s name |
| 100 | do |
| 104 | alldone |

Girl 1

| Age in weeks | First emergence of the word |
| --- | --- |
| 62 | alldone |
| 70 | no |
| 75 | okay |
| 76 | yes |
| 81 | mine |
|  | rock (doll’s crib) |
| 83 | child’s own name |
| 85 | go |
| 87 | you |
|  | love |
|  | I |
|  | cry |
|  | do |
| 91 | get back |
|  | know |
|  | nine |
| 95 | pray |
|  | make |
|  | take |
|  | better |
|  | cheer |
| 100 | me |
|  | one |
|  | two |
|  | three |
|  | five |
|  | six |
|  | ten |
| 102 | like |
|  | eat |

Girl 2

| Age in weeks | First emergence of the word |
| --- | --- |
| 63 | no |
| 66 | two |
|  | yes |
| 74 | me |
|  | one |
|  | three |
|  | five |
| 79 | alright |
|  | eight |
|  | nine |
| 83 | ten |
|  | I |
|  | seven |
|  | walk |
| 85 | help |
|  | sleep |
|  | hit |
| 87 | need |
| 88 | done |
|  | first |
|  | go |
|  | love |
|  | burp |
|  | kiss |
| 90 | happy |
| 93 | sing |
|  | wake up |
|  | call |
|  | my |
|  | alldone |
| 95 | six |
|  | allright |
|  | get |
| 96 | drop |
|  | four |
|  | okay |
| 98 | feed |
|  | more |
|  | turn round |
|  | her |
|  | want |
|  | have |
|  | call |
| 100 | come |
|  | stop |
|  | hungry |
|  | Can’t |
|  | do |
| 103 | give |
|  | good |

Girl 3

| Age in weeks | First emergence of the word |
| --- | --- |
| 44 | yes |
| 48 | no |
| 64 | one |
| 66 | me |
| 72 | Own’s name |
| 74 | round |
| 76 | more |
|  | I |
|  | stir |
| 78 | my |
|  | cook |
| 80 | tickles |
|  | five |
| 82 | hug |
|  | drink |
|  | sing |
|  | peek |
|  | all done |
|  | happy |
| 84 | wake up |
|  | burp |
|  | worry |
| 86 | you |
| 88 | fix |
|  | turn |
|  | help |
| 90 | wait |
|  | running (nose) |
|  | stuck |
|  | sleep |
| 92 | two |
|  | hear |
|  | spin (puzzle) |
|  | hold |
|  | cut |
|  | stuck |
| 98 | put |
|  | feed |
|  | make |
|  | open |
|  | go |
|  | other |
|  | her |
| 100 | fall |
|  | come |
|  | fit |
|  | get |
|  | stay |
|  | allright |
|  | can |
|  | lay down |
|  | clap |
| 102 | she |
|  | take |
|  | eat |
|  | her |
|  | much |
|  | feel better |
|  | Can’t |
|  | play |
|  | Don’t |
|  | seat |
|  | know |

Girl 4

| Age in weeks | First emergence of the word |
| --- | --- |
| 59 | no |
| 61 | I |
| 63 | yes |
| 78 | get |
|  | give |
| 80 | don’t |
|  | know |
| 82 | say |
|  | okay |
| 88 | cook |
|  | mine |
| 90 | do |
|  | they |
|  | take |
|  | go |
| 92 | sit down |
|  | get up |
|  | done |
| 94 | help |
|  | want |
|  | me |
| 96 | allgone |
|  | Two |
|  | Four |
|  | Seven |
|  | Nine |
|  | Ten |
|  | Eleven |
| 99 | rock (baby) |
|  | own’s name |
|  | some |
| 100 | drink |
|  | touch |
|  | bite (apple) |
| 103 | found it |
|  | need |
|  | walk |

**Appendix 2**

Types and age of acquisition (in weeks) of the first ten words and size of overall productive vocabulary at 24 months for each child

Boy 1. Productive vocabulary at 24 months: 127 words (38 abstract)

| Age in weeks | First emergence of the word |
| --- | --- |
| 59 | mama |
| 65 | bottle |
| 65 | cup |
| 65 | bowl |
| 65 | ham |
| 67 | no |
| 69 | gnam gnam |
| 70 | this |
| 70 | hi |
| 70 | doll |

Boy 2. Productive vocabulary at 24 months: 126 words (39 abstract)

| Age in weeks | First emergence of the word |
| --- | --- |
| 53 | ball |
| 55 | apple |
| 57 | light |
| 61 | mama |
| 61 | grape |
| 63 | yes |
| 63 | hi |
| 63 | watch |
| 63 | eye (doll) |
| 63 | baby |

Boy 3. Productive vocabulary at 24 months: 40 (13 abstract)

| Age in weeks | First emergence of the word |
| --- | --- |
| 45 | bye |
| 48 | no |
| 54 | brush |
| 56 | mama |
| 56 | papa |
| 62 | bottle |
| 66 | alldone |
| 66 | bye bye |
| 68 | allgone |
| 72 | camera |

Boy 4. Productive vocabulary at 24 months: 51 (11 abstract)

| Age in weeks | First emergence of the word |
| --- | --- |
| 58 | dad |
| 63 | hi |
| 64 | hello |
| 64 | no |
| 64 | bottle |
| 65 | yes |
| 70 | this |
| 73 | done |
| 74 | that |
| 74 | down |

Girl 1. Productive vocabulary at 24 months: 76 (30 abstract)

| Age in weeks | First emergence of the word |
| --- | --- |
| 47 | hi |
| 60 | bye |
| 62 | baby |
| 62 | spider |
| 62 | alldone |
| 64 | dad |
| 64 | mama |
| 66 | open |
| 70 | no |
| 75 | roar |

Girl 2. Productive vocabulary at 24 months: 164 (50 abstract)

| Age in weeks | First emergence of the word |
| --- | --- |
| 57 | Glu glu |
| 63 | mama |
| 63 | no |
| 63 | baby |
| 63 | hi |
| 66 | brush |
| 66 | two |
| 66 | yes |
| 66 | ball |
| 67 | bye |

Girl 3. Productive vocabulary at 24 months: 219 (64 abstract)

| Age in weeks | First emergence of the word |
| --- | --- |
| 44 | mama |
| 44 | gnam |
| 44 | yes |
| 46 | ball |
| 48 | sock |
| 50 | no |
| 56 | night |
| 56 | baby |
| 56 | daddy |
| 56 | hallo |

Girl 4. Productive vocabulary at 24 months: 128 (37 abstract)

| Age in weeks | First emergence of the word |
| --- | --- |
| 53 | hi |
| 55 | thank you |
| 59 | no |
| 59 | ahm ahm |
| 61 | I |
| 63 | this |
| 63 | yes |
| 63 | mama |
| 69 | bottle |
| 69 | baby |

**Appendix 3**

Progression between the size of abstract vocabulary and size of productive vocabulary by age 24 months

Abstract     Overall vocabulary

11  -->  51    (Boy 4)

13  -->  40    (Boy 3)

30  --> 76     (Girl 1)

37  --> 128   (Girl 4)

38  --> 127   (Boy 1)

39  --> 126   (Boy 2)

50  --> 164   (Girl 2)

64  --> 219   (Girl 3)
